# Sleep Bruxism: A Narrative Review of Current Concepts, Mechanisms, and Clinical Implications

**DOI:** 10.1002/snz2.70046

**Published:** 2026-04-20

**Authors:** Ramesh Balasubramaniam, Daniele Manfredini, Guangzhao Guan

**Affiliations:** ^1^ Discipline Lead and Program Convenor in Oral Medicine UWA Dental School The University of Western Australia Western Australia Australia; ^2^ Dental and Maxillofacial Department Perth Children's Hospital Western Australia Australia; ^3^ Department of Medical Biotechnologies School of Dentistry University of Siena Siena Italy; ^4^ Department of Oral Diagnostic and Surgical Sciences University of Otago Dunedin New Zealand

**Keywords:** bruxism, masticatory muscle activity, sleep bruxism, teeth clenching, teeth grinding

## Abstract

Sleep bruxism is defined as “masticatory muscle activity during sleep that is characterized as rhythmic (phasic) or non‐rhythmic (tonic) and is not a movement disorder or a sleep disorder.” This comprehensive narrative review synthesizes current knowledge on the prevalence, risk factors, assessment, and management of sleep bruxism. It emphasizes the condition's multifactorial nature, encompassing genetic predisposition, central nervous system mechanisms, alterations in sleep architecture, and psychosocial influences, particularly individual differences in stress sensitivity and coping capacity. The review also discusses recent advancements in assessment techniques and critically evaluates management strategies, including behavioral interventions, occlusal appliances, and pharmacological management. It highlights the importance of individualized, interdisciplinary approaches to care, guided where possible, by identification of underlying contributing factors such as psychological stress, neurological disorders, respiratory disturbances, or medication use, reflecting the evolving understanding of this complex activity.

## 1 Introduction

Sleep bruxism is defined as ‘masticatory muscle activity during sleep that is characterized as rhythmic (phasic) or non‐rhythmic (tonic) and is not a movement disorder or a sleep disorder’ (Verhoeff et al. 2025). Sleep bruxism may be present as a physiological phenomenon or associated with underlying psychological and/or medical conditions, such as movement disorders, sleep disorders, neurological disorders, psychiatric disorders, side effects of medications, or adverse effects of recreational drug use, to name a few. Clinically, bruxism may occur with or without observable effects on the stomatognathic system, such as tooth wear, fractured restorations, temporomandibular joint or masticatory muscle pain (Kurup et al. 2025), which ultimately determines whether prevention or management of sleep bruxism and its associated consequences are necessary (Manfredini et al. 2016).

To advance our understanding of sleep bruxism, an international consortium of experts in 2025 revisited its definition. Sleep bruxism is now considered as a motor behavior rather than a disorder, which may be either a risk factor (linked to one or more negative health outcomes) or protective factor (linked with one or more positive health outcomes) or neither (neutral factor that is considered a harmless behavior without posing a risk or providing protection from a health outcome) (Lobbezoo et al. 2018). As such, being a behavior rather than a disorder, sleep bruxism cannot be a comorbidity. Additionally, the previously utilized unvalidated grading and classification system to assess the likelihood of sleep bruxism occurring has been discarded.

## 2 Prevalence

Current prevalence estimates of sleep bruxism remain, at best, approximately due to methodological limitations in existing studies. Contributing factors include variations in diagnostic criteria (self‐reported vs polysomnography (PSG)); differences in frequency, intensity, and type of muscle activity (tonic, rhythmic, or mixed); heterogeneous study populations; and confounding factors such as medication use and psychosocial influences. The prevalence of sleep bruxism varies widely across the lifespan. The reported prevalence has a large range of 1%–49% and varies across different age groups, whereby the rates appear highest in childhood and decrease with age without gender differences (Melo et al. 2019). For example, among children and adolescents, reported prevalence ranges from 3% to nearly 50%. This heterogeneity likely reflects differences in diagnostic criteria, study designs, and age groups, rather than true epidemiological variation (Gomes et al. 2018; Shahbour et al. 2022). On the other hand, prevalence estimates are generally lower, ranging between 1% and 15%, in adults (Wetselaar et al. 2021). The largest PSG‐based study reported prevalence rates of 5.5% when combining questionnaire and PSG data, 7.4% based on PSG alone, and 12.5% based on questionnaire alone, highlighting the impact of diagnostic approach (Kim and Shin 2024). In older adults, estimates range from 3% in some studies to 16.2% in a recent German cohort (Lavigne and Montplaisir 1994; Rauch et al. 2023). This apparent inconsistency may be explained by their methodological differences (Dal Fabbro et al. 2023).

## 3 Risk Factors for Sleep Bruxism

Accumulated scientific evidence spanning the last three decades has considered sleep bruxism to have a multifactorial etiology (Manfredini 2024). While psychological factors are often emphasized as key contributors, sleep bruxism is now understood to originate from complex multisystem physiological processes involving both the central and autonomic nervous systems (Ekman et al. 2025; Kato et al. 2001; Sakai et al. 2017). Previously held notions that attributed mechanical factors, such as occlusal discrepancies, as singular etiologic determinants have been refuted by contemporary insights (Lobbezoo 2025). While occlusal discrepancies and certain craniofacial morphologies were once considered primary causes of sleep bruxism, current evidence suggests they play only a minor or secondary role. Instead, these factors may act as modulators that influence the manifestation or severity of sleep bruxism in predisposed individuals, rather than serving as its underlying cause. Nevertheless, it is important to acknowledge that in some parts of the world more than half of dental practitioners still consider occlusion to be the primary etiological factor for sleep bruxism, highlighting the persistence of this outdated concept and the need for continued dissemination of contemporary evidence (Mungia et al. 2025). Table 1 outlines the known risk factors for sleep bruxism.

**TABLE 1:** Risk Factors for Sleep Bruxism.

Risk Factors	Odds Ratio	Prevalence Ratio	Study
**Peripheral Factors**			
Facial morphology	Possible		Souza et al. (2020)
Malocclusion	2.00–2.50		Castroflorio et al. (2016)
**Central Factors**			
Pathophysiological			
Caffeine	1.40		Ohayon et al. (2001)
Smoking	1.30–2.80		Ohayon et al. (2001); Castroflorio et al. (2017); Frosztega et al. (2022)
Alcohol	1.80–1.90		Ohayon et al. (2001); Castroflorio et al. (2017)
SSRI (paroxetine)	3.63		de Baat et al. (2021)
SNRI (duloxetine, venlafaxine)	2.16–2.28		de Baat et al. (2021)
Attention deficit hyperactivity disorder	2.94		Souto‐Souza et al. (2020)
Gastroesophageal reflux	6.60		Castroflorio et al. (2017)
Chronic migraine	3.80		Castroflorio et al. (2017)
Insomnia		2.8	Maluly et al. (2020)
Obstructive sleep apnea	1.80	2.7	Maluly et al. (2020); Ohayon et al. (2001)
Snoring	1.40–3.14		Prado et al. (2018); Ohayon et al. (2001)
MDMA, cocaine, amphetamines			
Psychosocial			
Anxiety	1.09–1.3		Chattrattrai et al. (2022); Ohayon et al. (2001)
Post‐traumatic stress disorder	0.91–1.82		Knibbe et al. (2022)

### 3.1 Psychological Factors

Sleep bruxism has been frequently associated with anxiety, depression, and heightened stress sensitivity (Deregibus et al. 2025). Elevated levels of urinary catecholamines (adrenaline, noradrenaline, dopamine) and salivary cortisol have been observed in both children and adults with sleep bruxism, reflecting increased sympathetic activity (Karakoulaki et al. 2015; Seraidarian et al. 2009). Animal studies suggest that the lateral hypothalamic area and central amygdala may mediate trigeminal system reactivity to stress (Mascaro et al. 2009). Although a direct causal link remains unclear, symptoms such as reassurance sensitivity, anxious expectation, and panic are commonly reported in bruxism patients (Polmann et al. 2019). Individuals with sleep bruxism often exhibit maladaptive, task‐oriented coping styles and Type A personality traits (Giraki et al. 2010; Karakoulaki et al. 2015; Major et al. 1999; Schneider et al. 2007). The literature on the psychosocial contribution to sleep bruxism is inconsistent, likely due to confounding by lifestyle and exogenous factors including alcohol, tobacco, caffeine, medications, and recreational drugs (Rintakoski et al. 2010; Winocur et al. 2003; Winocur et al. 2001). Nonetheless, these findings support the conceptualization of sleep bruxism as a centrally mediated, multidimensional phenomenon in which psychological stress, anxiety, and depression may serve as potential triggers (Lobbezoo and Naeije 2001; Winocur et al. 2011).

### 3.2 Lifestyle Factors

Among the lifestyle risk factors, questionnaire studies have found cigarette smoking to be associated with a slight increase in the risk for sleep bruxism (odds ratio = 1.9) (Lavigne et al. 1997). Although nicotine, whether from active smoking or secondhand exposure, stimulates central dopaminergic activity, which may underlie its effect on sleep bruxism, a recent study found that psychological distress positively mediated the relationship between tobacco smoking and sleep bruxism (Pollis et al. 2025). Alcohol is a central nervous system depressant known to induce euphoria and alleviate anxiety with low to moderate intake. The acute and excessive use of alcohol is associated with a decrease in concentrations of serotonin, opioids, and dopamine. Although the precise mechanism is unknown, alcohol is associated with a slightly increased risk of sleep bruxism (odds ratio = 1.8–1.9) (Castroflorio et al. 2017; Ohayon et al. 2001). Notably, the consumption of one or two standard drinks daily does not appear to elevate the risk of sleep bruxism, whereas the intake of more than four standard drinks may marginally increase this risk (Hartmann et al. 1987). Caffeine is a commonly consumed psychoactive stimulant in society. Sleep bruxism has been reported to significantly increase in individuals who consume at least six cups of coffee per day (Odds Ratio = 1.4) (Bastien et al. 1990; Ohayon et al. 2001). Of interest unlike coffee, black tea consumption did not increase the intensity of sleep bruxism in one study (Frosztega et al. 2023).

### 3.3 Medical and Pharmacological Factors

Chronic systemic inflammation has been implicated in various diseases and may contribute to the pathophysiology of sleep bruxism. Young, otherwise healthy individuals with sleep bruxism demonstrate elevated levels of 17‐hydroxycorticosteroids, C‐reactive protein, and fibrinogen (Michalek‐Zrabkowska et al. 2020). Phasic bruxism correlates with higher glucose levels, while mixed bruxism is associated with increased daytime sleepiness, reduced oxygen saturation, and elevated heart rate, suggesting underlying metabolic and neuroendocrine dysregulation potentially linked to sympathetic overactivity and stress (Michalek‐Zrabkowska et al. 2020). Sleep bruxism has also been associated with endothelial dysfunction and hypertension (Kanclerska, Wieckiewicz, Poreba, et al. 2022; Michalek‐Zrabkowska, Martynowicz, Wieckiewicz, et al. 2021). Elevated total oxidant status and oxidative stress index, markers of systemic inflammation, are more prevalent in bruxers. Renalase, an enzyme involved in blood pressure regulation, correlates with bruxism severity, and hypertension, appears to be an independent risk factor for increased bruxism episodes (Kanclerska, Wieckiewicz, Poreba, et al. 2022; Martynowicz et al. 2018). Severe cases also show greater nocturnal systolic blood pressure variability (Michalek‐Zrabkowska, Wieckiewicz, Gac, et al. 2021), and lower plasma sodium levels have been linked to increased bruxism intensity and nocturnal diastolic blood pressure (Kanclerska, Wieckiewicz, Szymanska‐Chabowska, et al. 2022). Study also showed that there may be a correlation between current sleep bruxism and gastroesophageal reflux disease‐related symptoms (Pollis et al. 2026).

Certain medications (e.g., selective serotonin reuptake inhibitors (SSRI), serotonin norepinephrine reuptake inhibitors (SNRI), haloperidol, amphetamines) and recreational drugs (e.g., 3,4‐methylenedioxy‐methamphetamine, cocaine, methamphetamine) are known to elevate the risk for bruxism; however, the overall quality of evidence is weak (Table 2). Some of the antidepressants in adult populations appear to have a positive association with sleep bruxism. These include duloxetine (OR = 2.2), paroxetine (OR = 3.6), and venlafaxine (OR = 2.3). Conversely, the use of citalopram, escitalopram, fluoxetine, mirtazapine, and sertraline showed no increased risk for sleep bruxism. Regarding anticonvulsants, only barbiturates demonstrated an association with sleep bruxism in children (OR = 14.7), while benzodiazepines, carbamazepine, and valproate exhibited no increased risk. Methylphenidate showed an association with sleep bruxism in adolescents (OR = 1.7) (Melo et al. 2018).

**TABLE 2:** Medications with Potential to Induce Sleep Bruxism (de Baat et al. 2021; George et al. 2021; Montastruc 2023).

Classes	Drugs
Selective serotonin reuptake inhibitors	• Fluoxetine • Sertraline
Antipsychotics	• Aripiprazole • Risperidone • Olanzapine
Selective norepinephrine reuptake inhibitors	• Atomoxetine • Duloxetine • Venlafaxine
Dopamine agonists	• Haloperidol • Chlorpromazine • Trifluoperazine • Perphenazine
Antihistamine	• Ketotifen
Stimulants	• Amphetamines • Methylphenidate
Opioids	• Methadone
Recreational drugs	• Methamphetamine • Methylenedioxymethamphetamine • Cocaine • Heroin

### 3.4 Sleep‐Related Factors

In addition to medications, sleep arousal, characterized by brief awakening with increased brain, autonomic, and muscular activity, is thought to be one of the sleep‐related factors for sleep bruxism. The association between sleep bruxism and sleep arousal dates back to 1968 and 1971 (Reding et al. 1968; Satoh and Harada 1973), with subsequent studies, including PSG and electrophysiology, confirming that nearly 80% of sleep bruxism episodes are linked to sleep arousals, particularly during the cyclic alternating pattern phase A (Carra et al. 2011; Huynh, Kato, Rompré, et al. 2006; Kato et al. 2003; Okeson et al. 1994). Despite this strong association, sleep arousals are not considered the sole cause of sleep bruxism; rather, they are regarded as a “permissive window” that facilitates rhythmic masticatory activity (RMMA), similar to the mechanism observed in periodic limb movements. Notably, studies have shown that sleep bruxism encompasses a range of repetitive masticatory activities during sleep, including RMMA, bracing, and other jaw‐related movements, rather than being limited to RMMA alone (Gastaldo et al. 2006).

The role of disordered breathing during sleep as a potential etiological factor in sleep bruxism has been widely discussed over the past decade. Theories on a possible temporal relationship between sleep bruxism and obstructive sleep apnea (OSA) have been explored (Carra et al. 2011; Manfredini, Guarda‐Nardini, Marchese‐Ragona, et al. 2015). The hypothesis is that sleep bruxism events may be triggered by respiratory changes during sleep, such as desaturation, respiratory effort‐related arousal, hypopnea, or apnea events. However, recent findings show that the temporal association between sleep bruxism and respiratory events is not linear and may vary depending on the type of apnea, suggesting a more complex interaction than a simple protective reflex (Colonna et al. 2022). Notably, there are several common clinical features of sleep bruxism and sleep disordered breathing. Moreover, management for OSA, including mandibular advancement appliance therapy, adenotonsillectomy, and continuous positive airway pressure (CPAP) therapy, has shown a significant reduction in sleep bruxism in the short‐term (Balasubramaniam et al. 2014). For example, snoring (OR = 1.4) and OSA (OR = 1.8) have been associated with a slight increase in the risk for sleep bruxism (Ohayon et al. 2001). A study examining 3‐ to 16‐year‐old snorers who underwent PSG found 59% with the sign of sleep bruxism, and those exhibiting sleep bruxism demonstrated higher apnea index, apnea‐hypopnea index (AHI), and rapid eye movement (REM) sleep AHI compared to non‐bruxers (Sheldon 2010). Another investigation involving PSG of 38 consecutive children with probable obstructive sleep apnea‐hypopnea syndrome (OSAHS) indicated that 92.1% of children diagnosed with OSAHS experienced excessive sleepiness (29.4%) and sleep bruxism (34.3%); among these, eight children with severe OSAHS were also reported to exhibit sleep bruxism (Gregório et al. 2008). Among adult patients with functional somatic syndromes and upper airway resistance syndrome (UARS), over 50% were reported to exhibit sleep bruxism based on subjective measures such as bed partner reports of tooth grinding and dentist observations of tooth wear, although these indicators have recognized limitations in reliability (Gold et al. 2003). Additionally, in a study involving 21 adult patients suspected of sleep‐disordered breathing, 54% of mild OSA patients and 40% of moderate OSA patients were diagnosed with sleep bruxism; however, definitive conclusions were limited by the small sample size and the absence of PSG for sleep bruxism evaluation (Sjöholm et al. 2000). Interestingly, experimental evidence on this subject presents inconsistent findings. In young sleep bruxism individuals without sleep‐related respiratory issues, significant breaths and a 1%–2% drop in oxygen saturation are often observed preceding the onset of sleep bruxism (Dumais et al. 2015; Khoury et al. 2008). In OSA patients, the frequency of sleep bruxism events correlates positively with the severity of AHI and oxygen desaturation events (Hosoya et al. 2014). Conversely, despite a majority of sleep bruxism events occurring shortly after OSA events, a substantial proportion of sleep bruxism events also precede OSA, with ≈19% of sleep bruxism events unrelated to OSA (Saito et al. 2014). As such, the coexistence of sleep bruxism and sleep disordered breathing may be, in fact, coincidental (da Costa Lopes et al. 2020). However, a recent study examined the link between sleep bruxism and OSA in relation to sleep architecture. In OSA patients with sleep bruxism, there was a higher proportion of REM sleep, an 8‐fold lower AHI during REM sleep, and a lower arousal threshold. RMMA was higher in OSA patients with sleep bruxism, and the frequency of oromotor events correlated positively with AHI, emphasizing a unique OSA phenotype with distinct associations between respiratory events and RMMA, and non‐specific masticatory muscle activity (Okura et al. 2023). While plausible in a subgroup of the sleep bruxism population, particularly those with identified vulnerability or genetic predisposition such as retrognathia, large tonsils, deep palate, macroglossia, and high body mass index, the precise role of breathing in the etiology or genesis of sleep bruxism remains an enigma (De Luca Canto et al. 2014). Hence, to date, there is no evidence to support a conclusive temporal relationship between sleep bruxism and sleep disordered breathing (da Costa Lopes et al. 2020; Pauletto et al. 2022). The relationship between sleep bruxism and OSA is complicated by interindividual differences (Manfredini, Guarda‐Nardini, Marchese‐Ragona, et al. 2015). Hence, it is postulated that investigation of shared phenotypes between subjects with sleep bruxism and patients with sleep disordered breathing may ultimately reveal an underlying relationship.

### 3.5 Genetic and Familial Factors

Evidence for a genetic predisposition to sleep bruxism remains limited but suggestive. Family history is reported in 20%–50% of affected individuals, and childhood bruxism often persists into adulthood (Hublin et al. 1998). A cohort study incorporating PSG‐based RMMA assessment found that 37% of patients had at least one first‐degree relative with sleep bruxism, with relative risk ratios of 2.9 in survey data and 4.6 in sleep laboratory evaluations (Khoury et al. 2016). A Finnish twin study demonstrated higher concordance in monozygotic versus dizygotic twins, supporting a heritable component (Hublin et al. 1998). However, no specific inheritance patterns or causative gene polymorphisms have been confirmed. The fluctuating nature of sleep bruxism, with periods of remission and activity, complicates genetic phenotyping (Khoury et al. 2016). Some studies have implicated serotonergic genes. The HTR2A polymorphisms rs6313 (C allele; OR = 4.3) and rs2770304 (C allele; OR = 2.1) were associated with increased risk (Abe et al. 2012; Oporto et al. 2016), though serotonin's broad role in mood, pain, and sleep limits specificity. Additional associations have been found with matrix metalloproteinase‐9 and catechol‐O‐methyltransferase (Vieira et al. 2020). Environmental, occupational, and age‐related comorbidities likely influence expression (Abe et al. 2012; Lobbezoo et al. 2014). Nonetheless, twin studies in young adults suggest a substantial genetic contribution to phenotypic variability in sleep bruxism, with no observed sex difference (Rintakoski et al. 2012).

The understanding of the neurophysiology of sleep bruxism hinges on first accepting that sleep bruxism is a ‘sign’ rather than a disorder per se, similar to other clinical signs such as fever, erythema, and swelling (Manfredini et al. 2024). As with other clinical signs, sleep bruxism may be physiological when mild or pathophysiological when severe. It can be benign, transient, and within normal physiological limits, or it can become frequent and intense, exceeding an individual's adaptive capacity. In the latter case, sleep bruxism is considered pathophysiological, often associated with an underlying condition or disorder and may lead to clinically significant consequences (Manfredini et al. 2024).

## 4 Assessment and Detection of Sleep Bruxism

The assessment for sleep bruxism can be based on i) self‐report by the patient (or observed by a bed partner or parent), ii) the clinical examination, and/or iii) measurement by a device, either ambulatory or a monitored PSG (Verhoeff et al. 2025). In routine clinical practice, the assessment involves a comprehensive history and clinical examination to identify characteristic signs and symptoms, such as tooth wear or mobility, masseter hypertrophy, tongue indentations, dental hypersensitivity, masticatory muscle pain or fatigue during function, and temporomandibular joint clicking or locking, as well as to evaluate potential underlying conditions or risk factors, in line with the Standardized Tool for the Assessment of Bruxism (STAB) criteria (Manfredini et al. 2023). While polysomnographic recordings in a sleep laboratory could identify sleep bruxism events (Lavigne et al. 1996; Lavigne, Rompré, Poirier, et al. 2001), they do not fully quantify masticatory muscle activity such as jaw bracing (Manfredini et al. 2019). EMG recordings alone may often be sufficient for calculating event frequency, and recent studies have highlighted the importance of evaluating muscle work in sleep bruxism assessment (Colonna et al. 2025; Colonna et al. 2020; Colonna et al. 2022). In addition, alternative methods that do not require full PSG monitoring have been developed to assess masticatory muscle activity during sleep in the home environment (Thymi et al. 2021; Yamaguchi et al. 2023). While these instrumental approaches are valuable, standardized questionnaires, clinical history, and basic clinical assessments remain more practical and feasible for routine clinical practice (Paesani et al. 2013).

### 4.1 Subjective‐Based Assessment

The subjective‐based assessment consists of sleep and awake bruxism reports and the patient's complaints. The sleep bruxism report is based on the Oral Behaviors Checklist (Markiewicz et al. 2006; Ustrell‐Barral et al. 2025). The awake bruxism questionnaire is designed to elicit details about an individual's behaviors over the past month, including teeth grinding, teeth clenching, tooth contact, and jaw bracing. Although this review primarily focuses on sleep bruxism, assessment of awake bruxism may provide useful contextual information, as daytime and sleep‐related masticatory muscle activities may co‐occur and could reflect shared behavioral or neurophysiological mechanisms. Increasing evidence suggests that these two conditions frequently overlap in clinical populations (Teichert Filho et al. 2025). Patients often present reporting sleep bruxism based on observations from bed partners; however, studies using Ecological Momentary Assessment have demonstrated that awake bruxism behaviors are also commonly identified in individuals who report sleep bruxism (Manfredini et al. 2025). This overlap has important clinical implications, as clinicians should consider screening for awake bruxism when evaluating patients with suspected sleep bruxism to ensure a more comprehensive assessment and appropriate management. In terms of diagnostic accuracy, self‐reported sleep bruxism has demonstrated the highest sensitivity compared to morning jaw symptoms and EMG activity (Yachida et al. 2016). Notably, self‐report questionnaires and clinical signs displayed moderate sensitivity, specificity, and accuracy in diagnosing bruxism when contrasted with an ambulatory device used in sleep studies (Ohlmann et al. 2022). Moreover, various aspects, including headache, tooth wear, tinnitus, xerostomia, and drooling, are systematically examined using different evaluation tools such as the DC/TMD Symptoms Questionnaire, Tooth Wear Evaluation System (TWES) (Wetselaar and Lobbezoo 2016), xerostomia (explored via an item from the Xerostomia Inventory) (Thomson et al. 1999), and drooling (assessed using the Radboud Oral Motor Inventory for Parkinson, ROMP) (Kalf et al. 2011). These assessments provide a comprehensive overview of the patient's symptoms and contribute to a more thorough understanding of their overall oral health.

### 4.2 The Clinical Assessment

**FIGURE 1:**
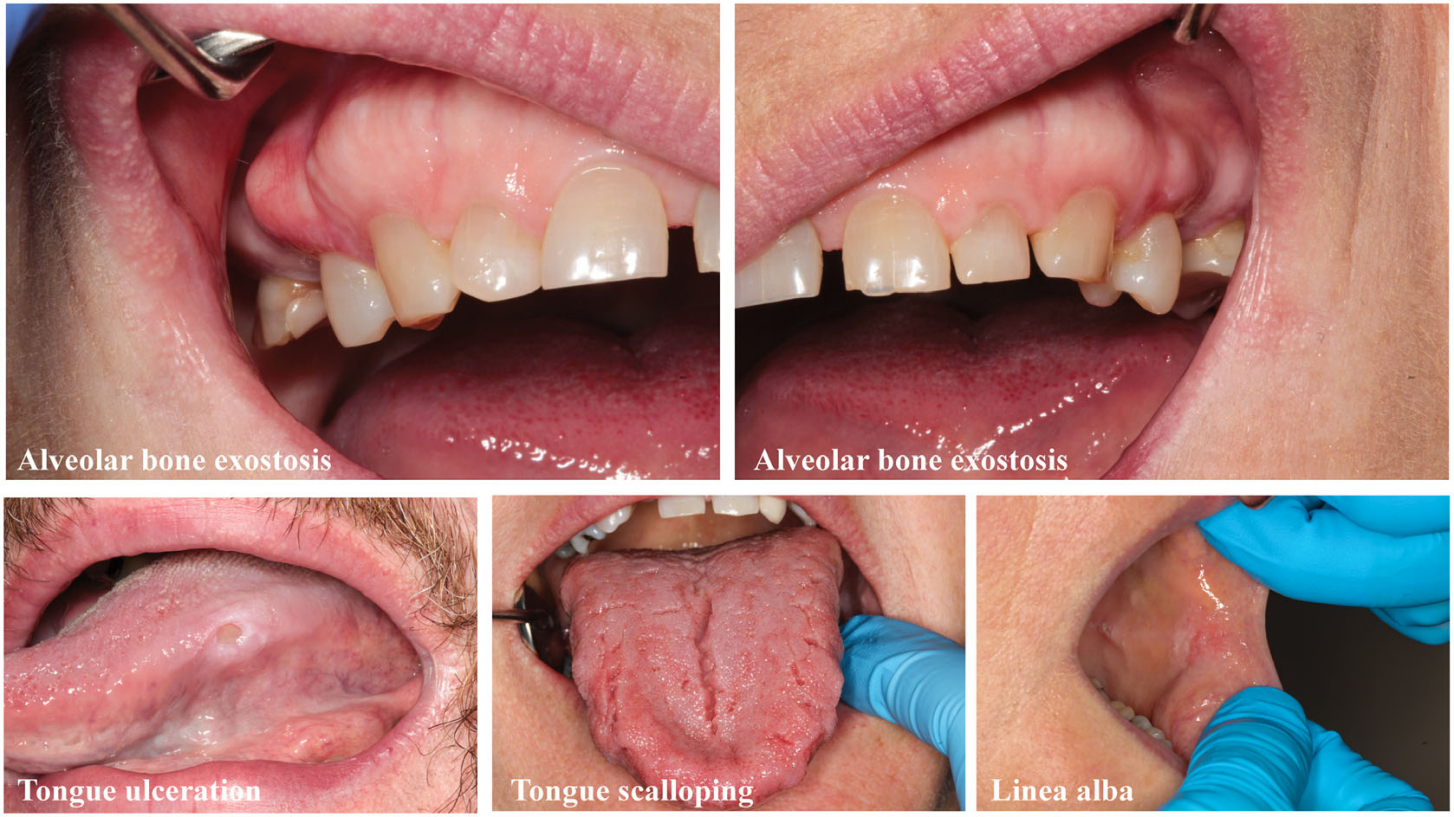
Intraoral presentation which may be observed in individuals with sleep bruxism.

The clinical assessment comprises both extraoral and intraoral examinations. The extraoral examination specifically targets the temporomandibular joints and the muscles of mastication. Evaluation of the temporomandibular joint encompasses an assessment of its range of movement, palpation for tenderness or pain, detection of sounds such as clicking or crepitation, and examination for locking or dislocation. When assessing the muscles of mastication, attention is given to identifying any discomfort or pain with masticatory muscle palpation and with function. A systematic approach is crucial to thoroughly examining all intra‐oral structures, especially recording details such as linea alba, lip impressions, tongue scalloping, tongue ulceration, and alveolar bone exostosis, to provide a comprehensive evaluation of bruxism (Yap and Chua 2016) (Figure 1). Within the context of this evaluation domain, the position of the tongue is assessed using the modified Friedman score (Friedman et al. 2013). Tooth wear screening, periodontal and dental examination, restoration, and oral appliance evaluation are important aspects of sleep bruxism evaluation. Additionally, piezoelectric film‐based intra‐occlusal splint recording devices have been employed for comprehensive documentation of bruxism activity (Baba et al. 2003; Takeuchi et al. 2001).

### 4.3 Instrument‐Based Assessment

The assessment of nocturnal masseter muscle activity often relies on widely used portable EMG recording systems, which exhibit a remarkable level of diagnostic accuracy in detecting sleep bruxism (Kudo et al. 2025; Yamaguchi et al. 2023). However, a limitation of these methods is their inability to determine whether a subject is genuinely asleep. Masseter muscle activity may be from daytime activities such as clenching, speaking, or eating. Hence, EMG of masticatory muscles performed through PSG, complemented by audio‐video recording (PSG‐AV) during an overnight stay in a sleep laboratory, is likely the most accurate method to assess sleep bruxism (Lavigne et al. 1996; Lobbezoo et al. 2013; Sateia 2014). Despite its effectiveness, the complexity and elevated cost associated with PSG‐AV have hindered its widespread integration into routine clinical practice for sleep bruxism assessment. To offer a cost‐effective alternative to inpatient PSG, modified ambulatory PSG devices (these devices typically include sensors and electrodes to monitor various physiological parameters, such as electroencephalogram, electro‐oculogram, electromyogram, electrocardiogram, respiratory effort, airflow, and oxygen saturation) were designed for detecting sleep bruxism. Unlike inpatient PSG, the ambulatory devices allow assessment in the individual's home environment, preserving their natural sleep setting. However, a drawback of ambulatory PSG is the absence of audio‐video recordings; hence, they are unable to detect tooth grinding sounds and jaw movements related to sleep bruxism. Consequently, distinguishing between tooth clenching and tooth grinding during sleep, as well as other oromotor activities, is not possible (Doering et al. 2008). The evaluation of PSG should be contingent upon assessing both arousal‐related and unrelated sleep bruxism events (Lavigne et al. 1996). To support this assessment, a range of tools may be used, including the bruxism episode index, masseter EMG, tooth wear index, self‐report bruxism questionnaires, and, where available, polysomnographic bruxism index (Table 3).

**TABLE 3:** Bruxism Indices.

Bruxism Indices	Measurement
**Bruxism episode index**	Measurement of the number of bruxism episodes per hour of sleep (<2: irrelevant SB; 2–4: mild/moderate SB; >4: severe SB) (Cartwright 2014)
**Masseter electromyography**	Measurement of the masticatory muscle's functionality (Ginszt and Zieliński 2021)
**Tooth wear index**	Clinical measurement of tooth wear. e.g. Smith and Knight tooth wear index (Smith and Knight 1984)
**Self‐report bruxism questionnaires**	History on sleep bruxism. e.g. standardised tool for assessment of bruxism (Manfredini et al. 2023)
**Polysomnographic bruxism index**	Recording of physiological events throughout an entire night of sleep utilizing electroencephalography, electro‐oculography, and electromyography (Lavigne et al. 1996)

Abbreviation: SB, sleep bruxism.

**FIGURE 2:**
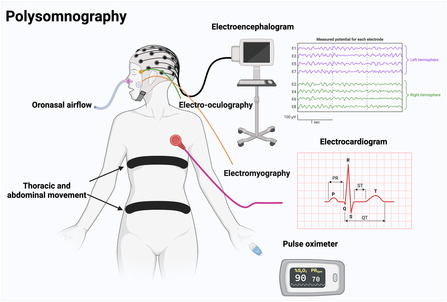
Polysomnography is classified into various levels (I–IV). A type I study typically includes an electroencephalogram, electro‐oculogram, electromyogram, electrocardiogram, nasal/oral airflow, thoracic and abdominal respiratory effort belts, a pulse oximeter, a snore microphone, body position sensors, leg movement sensors, and video monitoring for visual observation of sleep‐related behaviors. Created with BioRender.com.

While PSG is not mandatory for the clinical diagnosis of sleep bruxism, it may provide valuable information, particularly in research settings or when coexisting sleep disorders are suspected. The American Academy of Sleep Medicine recommends masseter EMG with synchronized audio‐video recording, though PSG has limited sensitivity due to night‐to‐night variability and is best reserved for select cases (Figure 2). Historically, devices for the assessment of bruxism incorporated ECG Holter monitoring because sleep bruxism events were considered to be associated with transient increases in heart rate. However, more recent evidence suggests that ECG monitoring may not always be necessary. A recent study demonstrated that EMG devices assessing the activity of a single masseter muscle without ECG monitoring detected nearly the same number of sleep bruxism events, with ≈99% overlap between the two protocols (Saracutu et al. 2025). In parallel with these developments, several non‐PSG devices are available, each with advantages and limitations. Bruxoff (Yanez‐Regonesi et al. 2023) shows moderate sensitivity and specificity, while Bitestrip (Shochat et al. 2007) offers compact EMG‐based screening with fair predictive value. GrindCare (Lobbezoo et al. 2020) provides ambulatory EMG monitoring with contingent electrical stimulation but lacks strong evidence‐based guidelines, and Dia‐Bruxo (Colonna et al. 2022) allows 24‐hour EMG recording for broader muscle activity analysis. Sunrise (Martinot et al. 2021) uses AI to detect mandibular movements, though it may miss clenching. While these devices could complement clinical evaluation, they primarily capture motor activity and cannot reliably distinguish bruxism phenotypes without audiovisual or clinical correlation (Li et al. 2024). In addition, current EMG‐based devices generally cannot differentiate between awake and sleep bruxism events without appropriate contextual information regarding the individual's sleep–wake cycle. Accurate identification of bedtime and waking time is therefore essential when assessing sleep bruxism using ambulatory EMG recordings, as even minor discrepancies in defining these time periods may substantially affect the number of detected events and the precision of the recorded data. Consequently, these devices should be considered adjunctive tools and should not replace comprehensive diagnostic approaches (Takahashi et al. 2026).

## 5 Management of Sleep Bruxism: Addressing Consequences and Behavior

Most individuals with sleep bruxism do not require active management, as the activity is often asymptomatic and considered non‐pathological behavior when no clinical consequences are present (Manfredini et al. 2020; Manfredini et al. 2024). Importantly, a clear distinction must be made between managing the consequences of bruxism and attempting to treat the bruxism activity itself. If sleep bruxism is a sign of an underlying disorder, management of the disorder should be prioritized where possible. At present, no intervention has been proven to eliminate sleep bruxism per se. Therefore, current management strategies primarily focus on mitigating the consequences, such as tooth wear, restoration damage, or orofacial pain, rather than eliminating bruxism behavior. Management of sleep bruxism approaches can be broadly categorized into: (i) behavioral medicine strategies, (ii) pharmacological interventions, and (iii) oral appliance therapy (Manfredini, Ahlberg, Winocur, et al. 2015; Minakuchi et al. 2022). Management, in contrast, refers to interventions that reduce the frequency or intensity of bruxism activity. This is considerably more challenging, as bruxism often has multifactorial origins. Successful management usually involves addressing the underlying cause, which may include psychological stress, psychiatric conditions, neurologic disorders, or medication side effects. Cognitive‐behavioral therapy, stress management techniques, or management of comorbid sleep and mental health disorders may reduce bruxism activity in select cases. Pharmacologic therapies have been investigated, but evidence supporting their sustained efficacy remains limited.

### 5.1 Behavioral Medicine

Behavioral medicine offers select strategies for managing sleep bruxism, although evidence remains limited (Table 4). A growing area of interest is the role of biofeedback and awareness training during wakefulness, which may influence sleep bruxism through improved self‐monitoring and modulation of daytime oral behaviors. Biofeedback therapy employs positive reinforcement to help patients acquire skills in reducing tension. The underlying principle is that individuals with sleep bruxism have the capacity to unlearn their habitual behavior (Cherasia and Parks 1986; Jokubauskas and Baltrušaitytė 2018). While some studies suggest that biofeedback techniques may be advantageous in diminishing both the frequency and intensity of sleep bruxism episodes, the underlying concept is that individuals may be able to identify and modify their physiological responses (Bergmann et al. 2020; Gu et al. 2015). Similarly, utilizing an occlusal splint with vibration stimulation presents an innovative strategy for addressing sleep bruxism. This therapeutic approach integrates vibratory stimulation into the design of the occlusal splint, with the objective of influencing muscle activity to alleviate sleep bruxism events. Notably, short‐term management incorporating contingent electrical stimulation, a form of biofeedback modalities, has shown effectiveness in reducing motor activities associated with sleep bruxism (Nakazato et al. 2021; Watanabe et al. 2001). Nonetheless, there is a scarcity of evidence regarding its long‐term effectiveness. Subsequent longitudinal studies with larger sample sizes are necessary to ascertain the clinical application of biofeedback in the management of sleep bruxism (Ilovar et al. 2014; Wang et al. 2014). The efficacy of contingent electrical lip stimulation for the reduction of sleep bruxism events is particularly noteworthy, given that the stimulation was administered for only half of the sleeping period (Nishigawa et al. 2003). GrindCare, a commercially available product, monitors jaw muscle activity associated with bruxism and employs contingent electrical stimuli to mitigate the frequency of jaw‐muscle activities (Needham and Davies 2013).

**TABLE 4:** Behavioral Medicine for the Management of Sleep Bruxism.

Behavioral Medicine
Sleep hygiene
Biofeedback
Cognitive‐behavioral therapy
Stress management including counseling and psychotherapy
Custom fabricated oral appliances to protect teeth and associated structures
Other behavioral or complementary methods: acupuncture, Jacobsen Progressive Muscle Relaxation and hypnotherapy

The efficacy of cognitive behavioral therapy (CBT) for sleep bruxism has been studied. CBT is a therapeutic modality designed to recognize and alter problematic thoughts, emotions, and behaviors. This approach has been applied in the management of various sleep disorders, including sleep bruxism. Some studies suggest that CBT, particularly in the form of relaxation techniques and stress management, may be beneficial in reducing the frequency and severity of sleep bruxism episodes (Amorim et al. 2018; Clarke and Reynolds 1991; Ommerborn et al. 2007). However, it is essential to note that the research on CBT for sleep bruxism is limited, and more well‐designed studies are needed to establish its effectiveness. Other behavioral or complementary methods, such as acupuncture, lack comprehensive research (Blasco‐Bonora and Martín‐Pintado‐Zugasti 2017). Similarly, both Jacobsen Progressive Muscle Relaxation and hypnotherapy, which aim to reduce overall muscle tension and induce a state of relaxation, lack rigorous scientific evidence. Of note, the effectiveness of these techniques may differ from person to person, emphasizing the need for a personalized approach through guidance from healthcare professionals such as psychologists or therapists.

More broadly, sleep hygiene education and counseling, such as advising against alcohol, caffeine, and tobacco use before bedtime, are recommended for all patients, given the frequent association between sleep bruxism and sleep arousals (Guaita and Högl 2016). Observational studies have also linked self‐reported bruxism with lifestyle factors such as increased screen time and high sugar consumption (odds ratios greater than 2, respectively) and, as such, should be avoided before bedtime (Restrepo et al. 2021). Nevertheless, robust evidence for the effectiveness of these lifestyle interventions in modifying bruxism activity is still lacking.

### 5.2 Oral Appliance Therapy

Oral appliance therapy involves the use of an intraoral removable appliance fabricated from either hard acrylic, soft vinyl, or nylon that fits on either dental arch between the maxillary and mandibular teeth. Their primary function is to protect teeth and restorations from damage caused by excessive grinding and clenching forces. Beyond this protective role, occlusal appliances have also been proposed to function as a ‘crutch’ for the masticatory muscles and the TMJ, potentially altering neuromuscular activity and reducing mechanical loading during sleep bruxism episodes. Supporting this concept, recent ultrasonographic evidence suggests that splint therapy may contribute to reductions in masseter muscle thickness and elasticity, indicating possible changes in muscle function following appliance use (Yalcin and Aslan Ozturk 2025). Occlusal appliances significantly affect slow‐wave sleep and overall sleep quality in individuals with sleep bruxism, regardless of their design or material; however, they do not reduce the frequency of bruxism episodes in the long term (Ferreira et al. 2026; Macedo et al. 2007). The most extensively studied oral appliance design used for sleep bruxism is the stabilization splint. Stabilization splints are complete flat plane appliances with balanced contacts against all opposing teeth in maximum intercuspation. They may be fabricated on the maxillary or mandibular arch and may include canine ramps, which have been noted to decrease elevator muscle activity (Fitins and Sheikholeslam 1993). Stabilization splints decrease the number of sleep bruxism episodes in the short term; however, this effect is not sustained (Minakuchi et al. 2022).

Of interest, mandibular advancement appliances are bimaxillary appliances employed to treat OSA and snoring. These appliances are designed to advance and stabilize the mandible and tongue and consequently minimize upper airway dilator muscle activity during inspiration, lessen upper airway collapsibility, and expand the pharyngeal airway space (Jayesh and Bhat 2015). Mandibular advancement appliances may decrease the number of sleep bruxism episodes, related signs and symptoms including headache, and occlusal forces in the short term (Carra et al. 2013; Franco et al. 2011; Mainieri et al. 2014; Singh et al. 2015). However, mandibular advancement appliances may be less comfortable to use compared to stabilization splints. Hence, mandibular advancement appliance therapy is not recommended for the management of sleep bruxism; however, it may be recommended for patients diagnosed with snoring and/or OSA with coexisting sleep bruxism (Landry et al. 2006). Of relevance, CPAP, the first‐line treatment for sleep‐disordered breathing, has also been shown to be effective in reducing sleep bruxism in the short term (Li et al. 2023).

As the primary objective of an oral appliance is to protect teeth from damage during sleep bruxism, it is essential that these appliances are comfortable and easy for patients to use and clean. There are many design types with associated anecdotal claims regarding their efficacy without scientific studies to support their claims. As a rule, the primary objective of an oral appliance is to protect teeth from damage during sleep bruxism. Hence, designs that meet the objective of protecting teeth, however, carry an increased risk for adverse effects such as swallowing, aspiration, and bite changes and are not recommended. Moreover, sleep bruxism in children is often considered a physiological behavior that tends to decrease with age as the stomatognathic system matures. Consequently, in many cases, active intervention may not be necessary, particularly in children in the mixed dentition stage. Management is generally conservative and focuses on monitoring, reassurance, and addressing potential contributing factors when clinically indicated (da Costa et al. 2024).

### 5.3 Pharmacotherapeutics

Several medications have been investigated to manage sleep bruxism, but none have demonstrated a consistently significant impact, and all carry potential side effects (Manfredini, Ahlberg, Winocur, et al. 2015). The use of pharmacotherapeutics should be reserved for severe cases or for instances where a coexisting condition may benefit therapeutically.

Much of our understanding of the use of pharmacotherapeutics is based on case reports, although certain medications, such as clonazepam and clonidine, have been subject to randomized controlled trials (RCTs) (Table 5). RCTs have demonstrated the potential efficacy of clonazepam, a benzodiazepine, in sleep bruxism. The efficacy of clonazepam 1 mg at bedtime was examined in three clinical trials. A significant reduction in both the number of episodes per hour (frequency) and the bruxism index (intensity) was evident (Sakai et al. 2017; Saletu et al. 2010; Saletu et al. 2005). Similarly, clonidine, a potent alpha‐2 (α_2_) adrenergic agonist administered at a nightly dose of 0.3 mg, exhibited a tendency to decrease the frequency of bruxism events per hour compared to a placebo (Huynh, Lavigne, Lanfranchi, et al. 2006). However, three out of 16 study subjects reported experiencing prolonged morning hypotension. In a subsequent small RCT, the administration of clonidine at a nightly dose of 0.15 mg over three nights demonstrated a greater than 30% reduction when compared to the placebo and clonazepam (Sakai et al. 2017).

**TABLE 5:** Pharmacotherapeutics for the Management of Sleep Bruxism.

Medication	Dosage	Effectiveness and Comments	Side‐Effects
**Clonazepam**	1 mg at bedtime (Saletu et al. 2005)	Reduction in both the number of episodes per hour and the bruxism index	Tiredness Sleepiness Slowed reaction time
**Clonidine**	0.3 mg nightly (Carra et al. 2010)	Decrease the frequency of bruxism events per hour	Prolonged morning hypotension
	0.15 mg nightly (Sakai et al. 2017)	30% reduction in the relative frequency of RMMA	Dry mouth
**Gabapentin**	100–300 mg at bedtime (Madani et al. 2013)	Decrease in sleep bruxism, including the number of episodes per hour, average masseter electromyography activities, and mean episode duration	No reported adverse events
**Rabeprazole**	10 mg before dinner (Ohmure et al. 2016)	A notable decrease in the frequency of electromyographic bursts, RMMA episodes, and grinding noise	No reported adverse events
**L‐dopa**	The first dose 1 h before bedtime and the second, 4 h after the first one (100 mg L‐DOPA and 25 mg benserazide) (Lobbezoo, Lavigne, Tanguay, et al. 1997)	Attenuate sleep bruxism	Nausea Diarrhea
**Bromocriptine**	1.25 mg was increased up to the maximum dose 7.5 mg within the first 6 days, 7.5 mg was maintained for the next 8 days (Macedo et al. 2014)	20% to 30% decrease bruxism episodes per sleep hour, but this result was not replicated in another study	Palpitations severe Dizziness Nausea
**Botulinum toxin type A**	200 units (60 into each masseter and 40 into each temporalis) (Ondo et al. 2018)	Improve subjective bruxism and painful symptoms associated with sleep bruxism	Altered smile (cosmetic change), muscle atrophy; muscle weakness

In one RCT, gabapentin 100–300 mg at bedtime led to a notable decrease in sleep bruxism, including the number of episodes per hour, average masseter EMG activities, and mean episode duration, without reported adverse events (Madani et al. 2013). The administration of rabeprazole, a proton pump inhibitor, resulted in a decrease in the frequency of electromyographic bursts, RMMA episodes, and grinding noise (Ohmure et al. 2016). L‐dopa demonstrated an ability to attenuate sleep bruxism, along with a reduction in the variance of root–mean‐square values, indicating a normalization of associated EMG activity patterns (Lobbezoo, Lavigne, Tanguay, et al. 1997). A clinical trial showed bromocriptine for 2 weeks, resulting in a 20% to 30% decrease in bruxism episodes per sleep hour compared to placebo (Lobbezoo, Soucy, Hartman, et al. 1997). These findings were not replicated in another study where bromocriptine did not intensify or diminish sleep bruxism motor activity (Lavigne, Soucy, Lobbezoo, et al. 2001).

Injecting botulinum toxin type A into the temporalis and masseter muscles could offer relief to individuals with sleep bruxism and associated persistent masticatory myalgia, temporal headache, and tooth wear and damage (Alonso‐Navarro et al. 2011; Cruse et al. 2022; De la Torre Canales et al. 2017; Ivanhoe et al. 1997; Ondo et al. 2018; Shim et al. 2020; Shim et al. 2014; Tinastepe et al. 2015). The mechanism of action is postulated to involve the reduction of jaw‐muscle contraction intensity. Similar to oral appliances, botulinum toxin type A does not decrease the frequency of sleep bruxism events in most patients. While injecting botulinum toxin type A appears to be an effective management, its therapeutic benefit typically diminishes in 3–4 months, whereby repeat injections are often necessary. As such, the use of botulinum toxin in the long term for sleep bruxism must be weighed against the cost as well as the risk of muscle atrophy and development of antibodies to the toxin.

It is important to note that the effectiveness of these management strategies could vary between individuals and hence should be individualized based on the patient's presenting complaint, examination findings, and medical and psychosocial histories. Of note in a nationwide twin cohort study on self‐reported sleep bruxism and mortality between 1990 and 2020, it was reported that ‘bruxism does not kill,’ and hence, in many cases, it is merely a sign of an innocuous behavior that captures the attention of an alert dentist but, in reality, is a harmless sign that does not warrant treatment (Ahlberg et al. 2024).

Overall, while management strategies are effective in reducing the consequences of sleep bruxism and form the mainstay of clinical care, true ‘management’ of sleep bruxism is less commonly achievable and typically requires a multidisciplinary approach targeting the underlying etiological or associated factors. Clinically, sleep bruxism should be considered a behavior rather than a disorder, and intervention is warranted primarily when adverse outcomes occur, such as temporomandibular disorders, significant tooth wear, prosthodontic complications, or orofacial pain. Management may involve strategies to reduce bruxism activity, mitigate its clinical consequences, or address associated medical conditions, including obstructive sleep apnea or gastroesophageal reflux disease, which have been shown to influence bruxism frequency. This framework allows practitioners to individualize care and distinguish between physiological sleep bruxism and cases requiring clinical intervention.

## 6 Conclusion

This comprehensive narrative review explores the current understanding of sleep bruxism, a condition encompassing a range of masticatory muscle activities during sleep. It discusses its multifactorial mechanisms and highlights its associations with conditions such as sleep disorders and stress. The review also outlines the latest approaches to assessing sleep bruxism, emerging insights from polysomnographic studies, and clinical implications, emphasizing the importance of personalized management strategies and interdisciplinary care.

## Funding

The authors have nothing to report.

## Conflicts of Interest

The authors declare no conflicts of interest.

## Data Availability

All data have been presented in the article.
